# Improved Outcomes of Thermal Ablation for Colorectal Liver Metastases: A 10-Year Analysis from the Prospective Amsterdam CORE Registry (AmCORE)

**DOI:** 10.1007/s00270-022-03152-9

**Published:** 2022-05-18

**Authors:** Robbert S. Puijk, Madelon Dijkstra, Bente A. T. van den Bemd, Alette H. Ruarus, Sanne Nieuwenhuizen, Bart Geboers, Florentine E. F. Timmer, Evelien A. C. Schouten, Jan J. J. de Vries, Bram B. van der Meijs, Karin Nielsen, Rutger-Jan Swijnenburg, M. Petrousjka van den Tol, Kathelijn S. Versteeg, Birgit I. Lissenberg-Witte, Hester J. Scheffer, Martijn R. Meijerink

**Affiliations:** 1 Department of Radiology and Nuclear Medicine, Amsterdam UMC, location Vrije Universiteit, De Boelelaan 1118, 1081HV Amsterdam, The Netherlands; 2 Cancer Center Amsterdam, Amsterdam, The Netherlands; 3 Department of Radiology and Nuclear Medicine, OLVG, Amsterdam, The Netherlands; 4 Department of Radiology and Nuclear Medicine, Garvan Institute of Medical Research, Kinghorn Cancer Centre, Darlinghurst, Sydney, NSW Australia; 5 Department of Surgery, Amsterdam UMC, location Vrije Universiteit, Amsterdam, The Netherlands; 6 Department of Surgery, University Hospital Birmingham, Birmingham, UK; 7 Department of Surgery, Medical Center Leeuwarden, Leeuwarden, The Netherlands; 8 Department of Medical Oncology, Amsterdam UMC, location Vrije Universiteit, Amsterdam, The Netherlands; 9 Department of Epidemiology and Data Science, Amsterdam UMC, location Vrije Universiteit, Amsterdam, The Netherlands; 10 Department of Radiology and Nuclear Medicine, Noordwest Ziekenhuisgroep, location Alkmaar, Alkmaar, The Netherlands

**Keywords:** Colorectal liver metastases (CRLM), Microwave ablation (MWA), Radiofrequency ablation (RFA), Local tumor progression-free survival (LTPFS), Long-term oncological outcomes

## Abstract

**Background:**

To analyze long-term oncological outcomes of open and percutaneous thermal ablation in the treatment of patients with colorectal liver metastases (CRLM).

**Methods:**

This assessment from a prospective, longitudinal tumor registry included 329 patients who underwent 541 procedures for 1350 CRLM from January 2010 to February 2021. Three cohorts were formed: 2010–2013 (129 procedures [53 percutaneous]), 2014–2017 (206 procedures [121 percutaneous]) and 2018–2021 (206 procedures [135 percutaneous]). Local tumor progression-free survival (LTPFS) and overall survival (OS) data were estimated using the Kaplan–Meier method. Potential confounding factors were analyzed with uni- and multivariable Cox regression analyses.

**Results:**

LTPFS improved significantly over time for percutaneous ablations (2-year LTPFS 37.7% vs. 69.0% vs. 86.3%, respectively, *P* < .0001), while LTPFS for open ablations remained reasonably stable (2-year LTPFS 87.1% [2010–2013], vs. 92.7% [2014–2017] vs. 90.2% [2018–2021], *P* = .12). In the latter cohort (2018–2021), the open approach was no longer superior regarding LTPFS (*P* = .125). No differences between the three cohorts were found regarding OS (*P* = .088), length of hospital stay (open approach, *P* = .065; percutaneous approach, *P* = .054), and rate and severity of complications (*P* = .404). The rate and severity of complications favored the percutaneous approach in all three cohorts (*P* = .002).

**Conclusion:**

Over the last 10 years efficacy of percutaneous ablations has improved remarkably for the treatment of CRLM. Oncological outcomes seem to have reached results following open ablation. Given its minimal invasive character and shorter length of hospital stay, whenever feasible, percutaneous procedures may be favored over an open approach.

**Supplementary Information:**

The online version contains supplementary material available at 10.1007/s00270-022-03152-9.

## Introduction

Colorectal liver metastases (CRLM) develop in up to 50% of patients with colorectal cancer, unfortunately only one-fifth of these patients are eligible for curative local treatment [1–6]. Most consider surgical resection the golden standard in upfront resectable CRLM, however, the deep-rooted mantra that surgical resection is the only curative intent treatment option for CRLM seems no longer factual [1, 2, 4, 7]. Radiofrequency (RFA) and microwave (MWA) ablation have proved themselves to result in cure in selected patients and consequently became routine treatment options for smaller-size hepatocellular carcinoma (≤ 2 cm) and unresectable small (≤ 3 cm) CRLM [1, 4, 8–10] .

Thermal ablation can be performed via an open, laparoscopic or percutaneous approach. Laparoscopic ablation is increasingly being performed due to its minimal invasive character compared to ablations via laparotomy, and local control rates are reported to be comparable between the two approaches. [11] However, laparoscopic ablation is technically more demanding and requires a fairly high level of expertise, which is presumably the reason that it is not yet widely embraced worldwide [4, 12, 13]. The percutaneous approach is mainly preferred in patients whose comorbid conditions preclude surgery, for centrally located tumors otherwise requiring a major resection (parenchyma-sparing), or in patients with regional or local tumor progression after prior local liver treatment [14–18]. This minimally invasive percutaneous approach is known for its favorable safety profile with low major complications rates (1.3%–2.4%) [14, 19, 20] .

Thermal ablation procedures have developed rapidly in terms of a potential learning curve effect, extensively upgraded device specifications, optimization of anesthetic techniques, use of image guidance tools and image fusion software platforms for volumetric assessment of the ablation zone [6, 7, 21–26]. When it comes to analyzing the efficacy and improvement of a certain treatment modality, the technique to eradicate tumors can best be elucidated by analyzing local control and time-to-local tumor progression [2, 27]. Local tumor progression (LTP) rates after thermal ablation of CRLM vary widely in the literature, ranging 7.6–22.2% for patients treated by percutaneous procedures and 2.7–9.5% for patients treated by open ablation [7, 11, 28–32]. Median overall survival (OS) rates after thermal ablation are reported mainly in matched cohorts or after multivariable analysis and vary from 34.3 to 53.2 months with 5- and 10-year survival rates of 20.8–60.0% and 18.0%, respectively [9, 19, 20, 33–39] .

As oncological outcomes of thermal liver ablation differ substantially among semi-recently published papers and evidence regarding the potential improvement over time, in terms of local control and time-to-local tumor progression, is lacking, this single-center Amsterdam Colorectal Liver Met Registry (AmCORE) based study aimed to analyze local disease control and survival outcomes following thermal ablation in patients treated for hepatic metastases from colorectal cancer over the last 10 years.

## Material and Methods

### Patients

Data were sourced from a prospective, longitudinal tumor registry for patients with hepatic metastases from colorectal cancer. All patients were treated at the Amsterdam UMC, location Vrije Universiteit (Amsterdam, the Netherlands), a tertiary referral institution for hepatobiliary and gastrointestinal malignancies. Approval was granted from the affiliated Institutional Review Board (reference number 2021.0121).

**Fig. 1:**
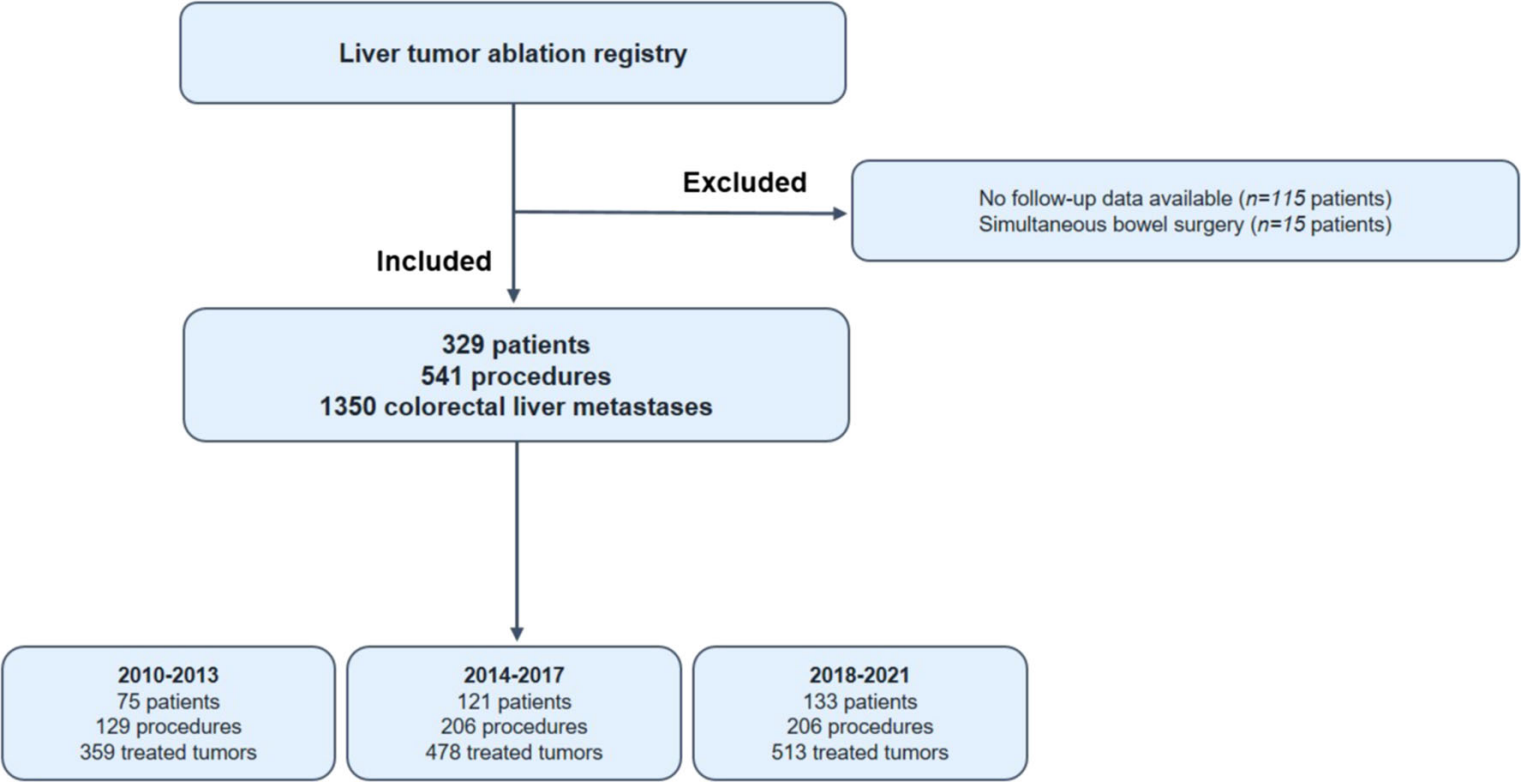
Flowchart of in- and excluded patients

Between January 2010 and February 2021, 449 consecutive patients with liver-only metastatic colorectal carcinoma underwent open or percutaneous thermal ablation with RFA or MWA (Fig. 1). One-hundred fifteen patients were excluded for having no available follow-up data at our institute. Although higher morbidity rates have never been reported after simultaneous liver ablation and bowel resection, partial hepatectomy plus colon surgery is known to be associated with a significant increased postoperative morbidity rates [40]. To overcome potential outcome interference, 15 patients were excluded having received simultaneous bowel resection. The remaining 329 patients underwent 541 procedures for 1350 liver metastases. Preprocedural treatment planning (e.g., angle of probe insertion) was performed prior to all procedures, and for percutaneous sessions, all needles/antennae were inserted under real-time computed tomography (CT) imaging. All patients had an Eastern Cooperative Oncology Group status of ≤ 2. The diagnosis of CRLM was based on cross-sectional imaging containing CT, magnetic resonance imaging (MRI) and [18F]-fluoro-2-deoxy-D-glucose (^18^F-FDG) positron emission tomography (PET)–CT scans. Treatment planning was routinely discussed in a multidisciplinary tumor board. An open rather than a percutaneous approach was chosen in case of liver metastases needing concomitant partial hepatectomy or when a percutaneous approach was technically not feasible due to the position of the tumor (e.g., in close proximity to the stomach). Although induction systemic therapy is not standard of care within the Netherlands, three patient categories did often receive induction systemic therapy first, namely: (A) patients with locally advanced primary (rectal) cancer, (B) patients with unresectable but potentially downstagable CRLM or with difficultly resectable disease if systemic therapy is likely to reduce procedural risk, and (C) patients with early metachronous disease. Chemotherapy regimen consisted of either capecitabine or irinotecan monotherapy, capecitabine and oxaliplatin (CAPOX), capecitabine + irinotecan (CAPIRI), folinic acid + 5-fluorouracil + oxaliplatin (FOLFOX) or folinic acid + 5-fluorouracil + irinotecan (FOLFIRI). Additional monoclonal antibodies (bevacizumab or panitumumab) were added in case of potentially downstagable disease. Conformal to national guidelines, no patients received adjuvant systemic therapy. [41].

The baseline characteristics of all enrolled patients are summarized in Table 1. Of 541 procedures, 232 were performed intraoperatively and 309 under CT guidance. A total of 653 metastases were treated with RFA (481 by open approach; 172 percutaneous) and 697 metastases with MWA (327 open and 370 percutaneous). A total of 171 procedures (31.6%) were performed after induction chemotherapy. The median number of treated tumors per procedure was 2.0 (IQR 3.0) in the entire cohort. Of 232 open procedures for 808 metastases, 449 (55.6%) metastases were ablated in the same session as concurrent partial hepatectomy was performed. Median follow-up time after each ablation was 16.5 months (IQR 26.8) in the entire cohort.

**Table 1:** Clinical characteristics

		Total	2010–2013	2014–2017	2018–2021	*P* value
*Patient-related characteristics*		*N* = 329	*N* = 75	*N* = 121	*N* = 133	
Gender	Male Female	222 107	52 23	86 35	84 49	.375 ^a^
Age, years*		65.3 (10.8)	63.4 (10.5)	65.5 (9.5)	66.2 (12.0)	.196 ^b^
ASA physical status	1 2 3 Unknown	23 229 71 6	7 53 12 3	9 86 25 1	7 90 34 2	.493 ^a^
Comorbidities	None Minimal Major Unknown	160 118 45 6	34 30 8 3	61 37 21 2	65 51 16 1	.449^a^
BMI (kg/cm^2^)*		26.0 (4.5)	25.7 (4.1)	26.2 (4.5)	25.9 (4.8)	.539^b^
*Disease-related characteristics*						
Clinical Risk Score (CRS)	0–2 ≥ 3 Unknown	139 92 98	31 17 27	39 36 46	69 39 25	.201^a^
Diagnosis of CRLM	Synchronous Metachronous Unknown	176 122 31	38 27 10	57 45 19	81 50 2	.653^a^
Primary tumor location	Right-sided Left-sided Rectum Unknown	89 145 93 2	25 23 25 2	29 55 37 –	35 67 31 –	.093^a^
RAS status	RAS wild type RAS mutation Unknown	29 22 278	6 3 66	7 5 109	16 14 103	.773^a^
BRAF V600 status	BRAF wild type BRAF mutation Unknown	46 3 280	7 1 67	11 – 110	28 2 103	.522^a^
MSS/MSI status	MSS MSI Unknown	58 1 270	6 0 69	16 0 105	36 1 96	.739^a^
*Procedure-related characteristics*		*N* = 541	*N* = 129	*N* = 206	*N* = 206	
Situation	Thermal ablation alone Simultaneous partial hepatectomy Simultaneous IRE	363 146 32	90 38 1	125 65 16	148 43 15	.006^a^
Induction chemotherapy	No Yes	370 171	80 49	153 53	137 69	.048^a^
No. of locally treated tumors	1–3 ≥ 4	374 167	83 46	148 58	143 63	.349^a^
Approach	Open Percutaneous	232 309	76 53	85 121	71 135	< .001^a^
Anesthesia technique	General anesthesia Midazolam + fentanyl sedation Propofol sedation Unknown	317 68 152 4	108 19 – 2	106 49 50 1	103 – 102 1	NA
Image guidance technique	Conventional (intraoperative US or CT fluoroscopy) CT hepatic arteriography	302 239	113 16	105 101	84 122	NA
Ablation modality	Radiofrequency RF3000™, LeVeen™ Cool-tip™ Starburst® (RITA®) Unknown RFA device Microwave Evident™ Solero™ Emprint™ with Thermosphere™ Unknown MWA device Unknown ablation modality	240 210 13 10 3 301 19 9 262 6 9	113 98 5 7 – 16 13 – – 2 4	120 112 5 8 – 86 12 5 66 2 1	7 – 3 – 4 199 2 4 188 2 4	< .001^a^
*Tumor-related characteristics*		*N* = 1350	*N* = 359	*N* = 478	*N* = 513	
Diameter, mm*		16.2 (11.5)	17.0 (12.9)	16.0 (11.8)	15.9 (10.1)	.686^b^
Size, mm	Small (1–30) Intermediate (31–50) Large (> 50) Unknown	1125 147 22 56	274 46 11 28	399 53 8 18	452 48 3 10	.010^a^

### Ablation Method

The vast majority of open and percutaneous ablations were performed by three interventional radiologists (BM, JV, MM) who have performed and/or supervised > 100 image-guided tumor ablations. The staff in our department has been almost stable over the last ten years. Approximately one-third of the procedures were performed by two interventional radiologists at the same time. During approximately 60% of all ablation procedures, the senior interventional radiologist (MM) was present. The procedure and other study-related details are given in supplementary materials (Appendix 1).

### Efficacy Evaluation and Follow-Up Strategy

Within the first two weeks after the initial procedure, a quality control contrast-enhanced CT scan was performed when there was a potential inadequate safety margin (0–5 mm) in combination with sub-optimal tumor conspicuity and needle visibility during the procedure [6]. This allowed for an early completion ablation procedure, if indicated. Follow-up should have consisted of at least one cross-sectional imaging modality study to reliable exclude or detect LTP. Regular follow-up consisted of [18]F-FDG-PET CT scans every 3 months after the initial ablation during the first year of follow-up and roughly every 6 months thereafter, according to national guidelines [41] and the standardization paper [2]. Additional MRI was only performed in case of uncertainty whether LTP was present. Follow-up imaging was reviewed by the interventional oncology team, certified diagnostic abdominal radiologists and nuclear physicians. If loco-regional disease recurrence was found on follow-up imaging, optimal retreatment was offered based on recommendations of the multidisciplinary team, depending on the extent of the disease in the liver, hepatic function, extrahepatic metastases and general condition of the patient.

### Data Collection and Statistical Analysis

For the sake of oncological outcome analyses, the entire cohort was divided into three subgroups (2010–2013, 2014–2017 and 2018–2021). Standard demographic, clinical and surveillance data were retrieved from the electronic database. Categorical variables are reported as frequencies (with or without percentage; %), whereas continuous variables are presented as median (IQR, interquartile range) or mean (± SD, standard deviation). Differences between the three subgroups in terms of baseline variables and outcomes were determined by using the Pearson Chi-square (χ2) test for categorical variables (^a^) and the one-way ANOVA (^b^) for comparison of means between the three subgroups.

Endpoint definitions were used along the consensus guidelines for the definition of time-to-event endpoints in image-guided tumor ablation by Puijk et al. [27] To study the primary endpoint, a time-to-event superiority analysis was used to analyze local tumor progression. LTP was defined as growth of tumor tissue at the initial treated tumor site [2, 27]. Patients were followed until the first recorded evidence of LTP (event) or until the last follow-up exam for those alive without LTP. Local tumor progression-free survival (LTPFS) curves, per patient and per tumor, were estimated using the Kaplan–Meier method and compared between subgroups using the log-rank test. Death without LTP was considered a competing risk. LTPFS over time was analyzed by allocating patients into one of three historical cohorts (2010–2013; 2014–2017 and 2018–2021). Baseline variables with *P*-values < .05 were entered in the univariable analysis. Uni- and multivariable analyses for LTPFS were performed by using the Cox proportional hazard regression model in the entire cohort. Variables with *P* < .05 in the univariable analysis were included in the final multivariable model. Hazard ratios (HR) and 95 percent confidence intervals (95% CI) were calculated. Using backward selection procedure, results of step-by-step removed variables were reported. Results are from last step before removal. Secondary endpoints were overall survival (OS) and safety. OS probability was estimated using the Kaplan–Meier method (time from the first ablation until the date of death or to the last follow-up visit or exam) for the entire cohort. Death during the index hospitalization or within 30 days after treatment was considered perioperative mortality. Safety in terms of complications was evaluated and reported using the standardized Common Terminology Criteria for Adverse Events (CTCAE) grading system, version 4.0 and 5.0. [2, 27, 42].

Statistical analyses were performed in consultation with an independent statistician (BLW) using SPSS® software, version 24.0 (IBM®, Armonk, New York, USA) [43] and the R software package, version 3.6.3 (R Foundation, Vienna, Austria) [44]. Statistical significance was established for *P* < .05. All results were reported according to the Strengthening the Reporting of Observational Studies in Epidemiology (STROBE) guidelines for reporting observational study data. [45].

## Results

### Technical Success and Local Tumor Progression

**Fig. 2:**
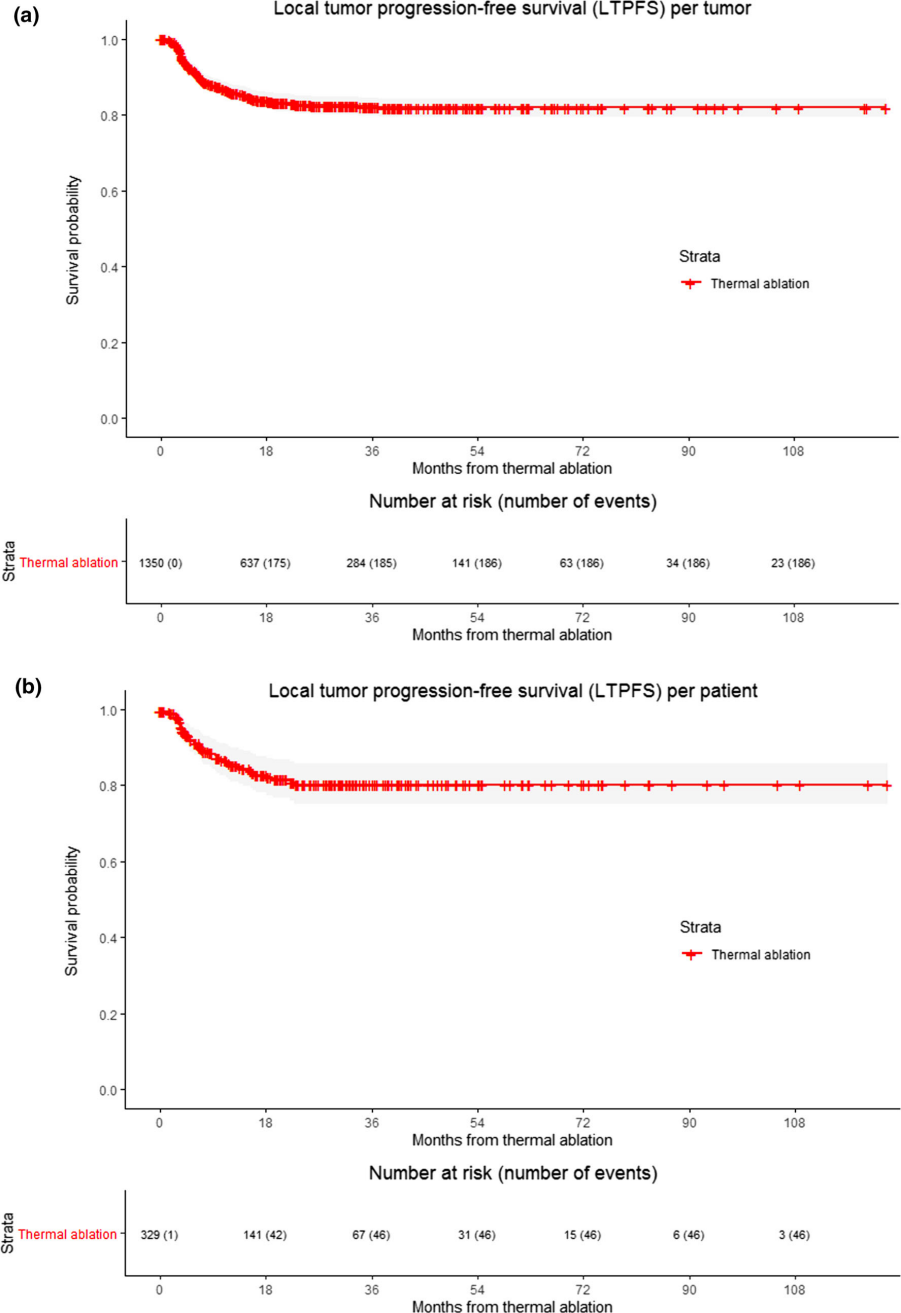
Kaplan–Meier survival curves indicating local tumor progression-free survival (LTPFS) per treated tumor (**A**) and per patient (**B**) after all thermal ablation sessions. Numbers at risk correspond to the amount of tumors and number of patients respectively. Death without local tumor progression (LTP) is censored (competing risk)

A total of 329 patients (mean age, 65.3 years ± 10.8; 222 men) met the inclusion criteria (Fig. 1 and Table 1). Incomplete ablation rate was 1.0% (14/1350), identified on early follow-up imaging and retreated within ten weeks following the initial ablation. The cumulative LTP rate after 6 months, 1, 2 and 3 years follow-up was 7.4% (100/1350), 11.6% (156/1350), 13.6% (183/1350) and 13.9% (186/1350), respectively, in the entire cohort demonstrated in Table 2 and illustrated as Kaplan–Meier estimates of LTPFS in Fig. 2. For small-size metastases only (≤ 3 cm) (*n* = 1125), the cumulative LTP rate was 10.7% (120/1125) during a median follow-up duration of 17.5 months (IQR 27.1).

**Table 2:** Outcomes of all thermal ablation procedures

		Total	2010–2013	2014–2017	2018–2021	*P* value
*Patient-related outcomes*		*N* = 329	*N* = 75	*N* = 121	*N* = 133	
Perioperative mortality (< 30 days)		1 (0.3%)	–	1	–	NA
*Procedure-related outcomes*		*N* = 541	*N* = 129	*N* = 206	*N* = 206	
Complications (CTCAE)	Grade 1 Grade 2 Grade 3 Grade 4 Grade 5 Missing	28 (5.2%) 38 (7.0%) 35 (6.5%) 5 (0.9%) 5 (0.9%) 13 (2.4%)	8 (6.2%) 6 (4.7%) 11 (8.5%) – 1 (0.8%) 5 (3.9%)	9 (4.4%) 21 (10.2%) 13 (6.3%) 4 (1.9%) 2 (1.0%) 4 (1.9%)	11 (5.3%) 11 (5.3%) 11 (5.3%) 1 (0.5%) 2 (1.0%) 4 (1.9%)	.404^a^
Follow-up, months, median (IQR)		13.1 (26.6)	10.6 (44.0)	18.6 (33.7)	11.5 (16.1)	< .001^b^
*Tumor-related outcomes*		*N* = 1350	*N* = 359	*N* = 478	*N* = 513	
Two-year LTP rate, no. tumors		183 (13.6%)	78 (21.7%)	72 (15.1%)	36 (7.0%)	< .001^a^
Time to detection of LTP, months, mean (SD)		7.1 (5.5)	5.8 (4.8)	8.4 (6.3)	6.9 (4.9)	.074^b^

Multivariable analysis revealed four factors associated with an inferior LTPFS (Table 3): no induction chemotherapy (HR 0.480, *P* < .001), percutaneous approach (HR 4.265, *P* < .001), larger size of metastasis (HR 1.932 for intermediate size [31-50 mm] and HR 4.783 for large size [> 50 mm], *P* < .001). Adjusted HR of ablations performed between 2014–2017 compared to 2010–2013 was 0.437 (95% CI 0.301–0.636) and 2018–2021 compared to 2010–2013 was 0.244 (95% CI 0.142–0.419) (*P* < .001).

**Table 3:** Factors associated with local tumor progression-free survival identified by univariable and multivariable Cox regression analyses from the time of the first intervention to local tumor progression

		Univariable analysis		Multivariable analysis	
		HR (CI)	*P*-value	HR (CI)	*P*-value
Time frame	2010–2013	Reference	** < .001**	Reference	** < .001**
	2014–2017	0.649 (0.471–0.894)		0.437 (0.301–0.636)	
	2018–2021	0.367 (0.247–0.545)		0.244 (0.142–0.419)	
*Procedure-related factors*					
Local treatment	Thermal ablation alone	Reference	** < .001**	Reference	.462
	Simultaneous partial hepatectomy	0.395 (0.272–0.574)		1.206 (0.725–2.007)	
	Simultaneous IRE	0.463 (0.217–0.989)		0.668 (0.290–1.543)	
Chemotherapy	No	Reference	** < .001**	Reference	** < .001**
	Yes	0.321 (0.228–0.453)		0.480 (0.332–0.694)	
Approach	Open	Reference	** < .001**	Reference	** < .001**
	Percutaneous	3.686 (2.722–4.990)		4.265 (2.747–6.622)	
Modality	RFA	Reference	**.026**	Reference	.855
	MWA	0.718 (0.535–0.963)		0.964 (0.648–1.434)	
*Tumor-related factors*					
Size of metastasis (mm)	Small (1–30)	Reference	** < .001**	Reference	** < .001**
	Intermediate (31–50)	2.536 (1.747–3.682)		1.932 (1.321–2.825)	
	Large (> 50)	8.436 (4.647–15.313)		4.783 (2.596–8.814)	

**Fig. 3:**
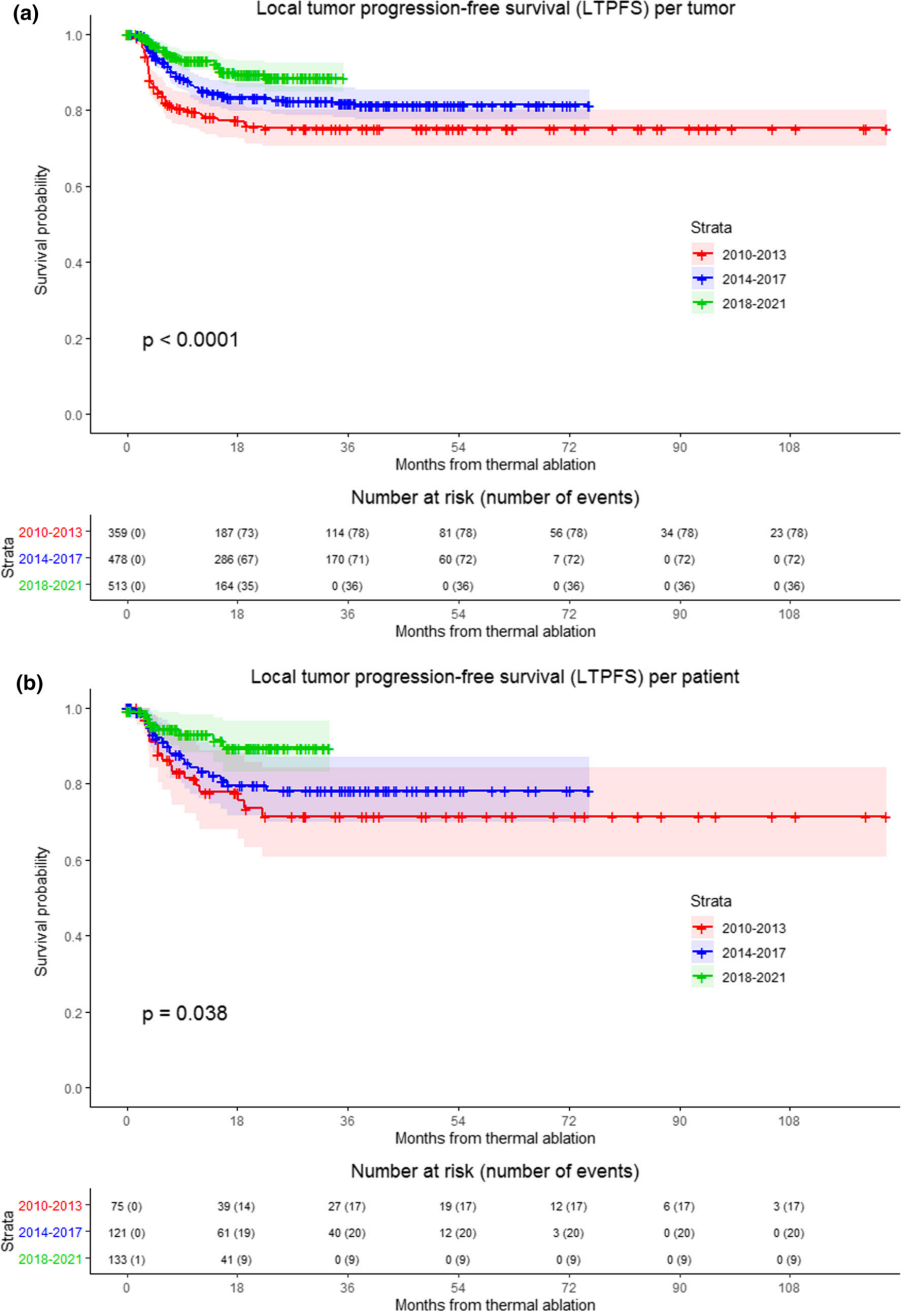
Kaplan–Meier survival curves indicating the local tumor progression-free survival (LTPFS) per time frame. Analysis per treated tumor (**A**) and per patient (**B**). Time frames: 2010–2013; 2014–2017 and 2018–2021. Numbers at risk correspond to the amount of tumors and number of patients respectively. Overall comparison log-rank (Mantel–Cox) test is reported per graph. Death without local tumor progression (LTP) is censored (competing risk).

**Fig. 4:**
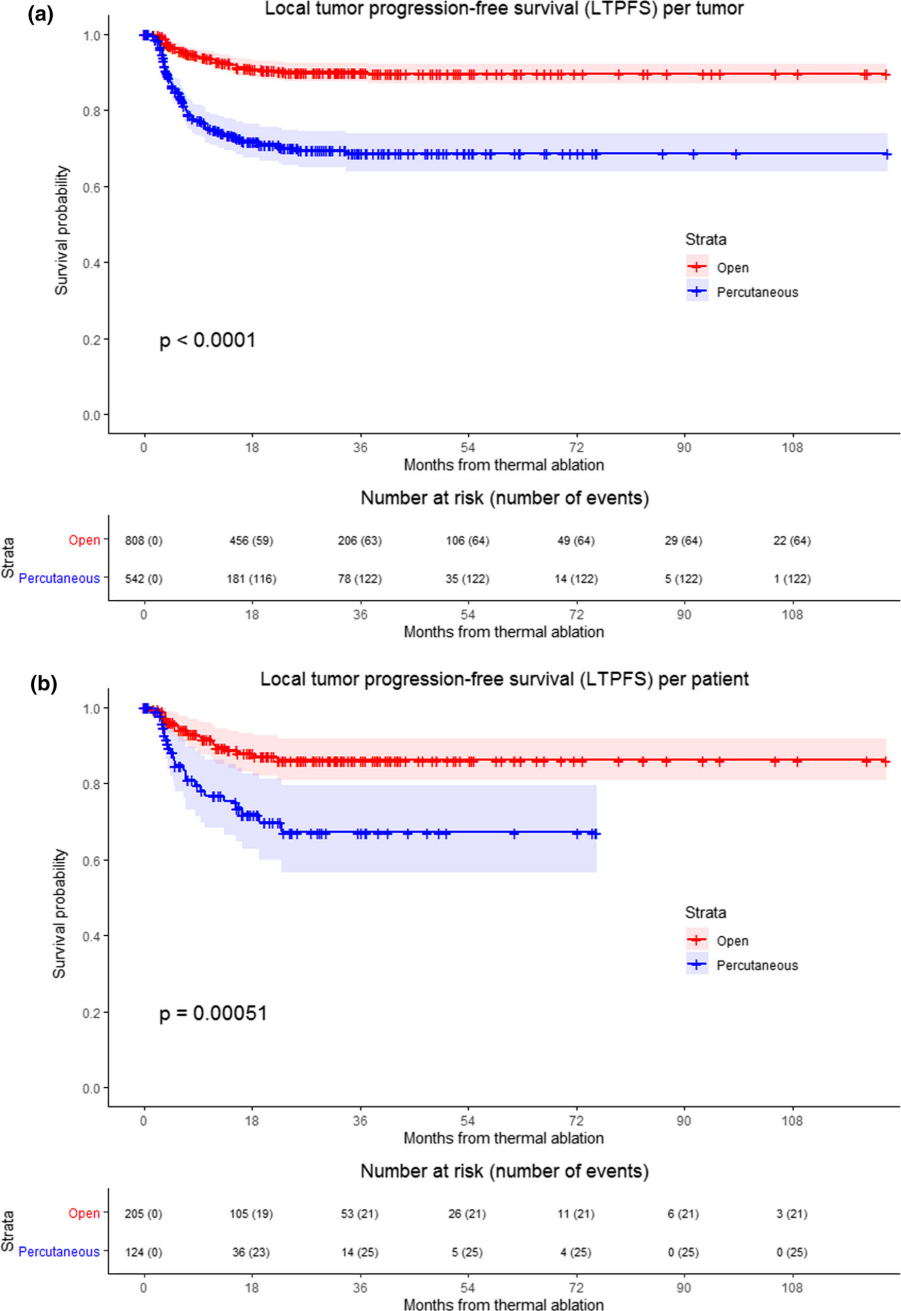

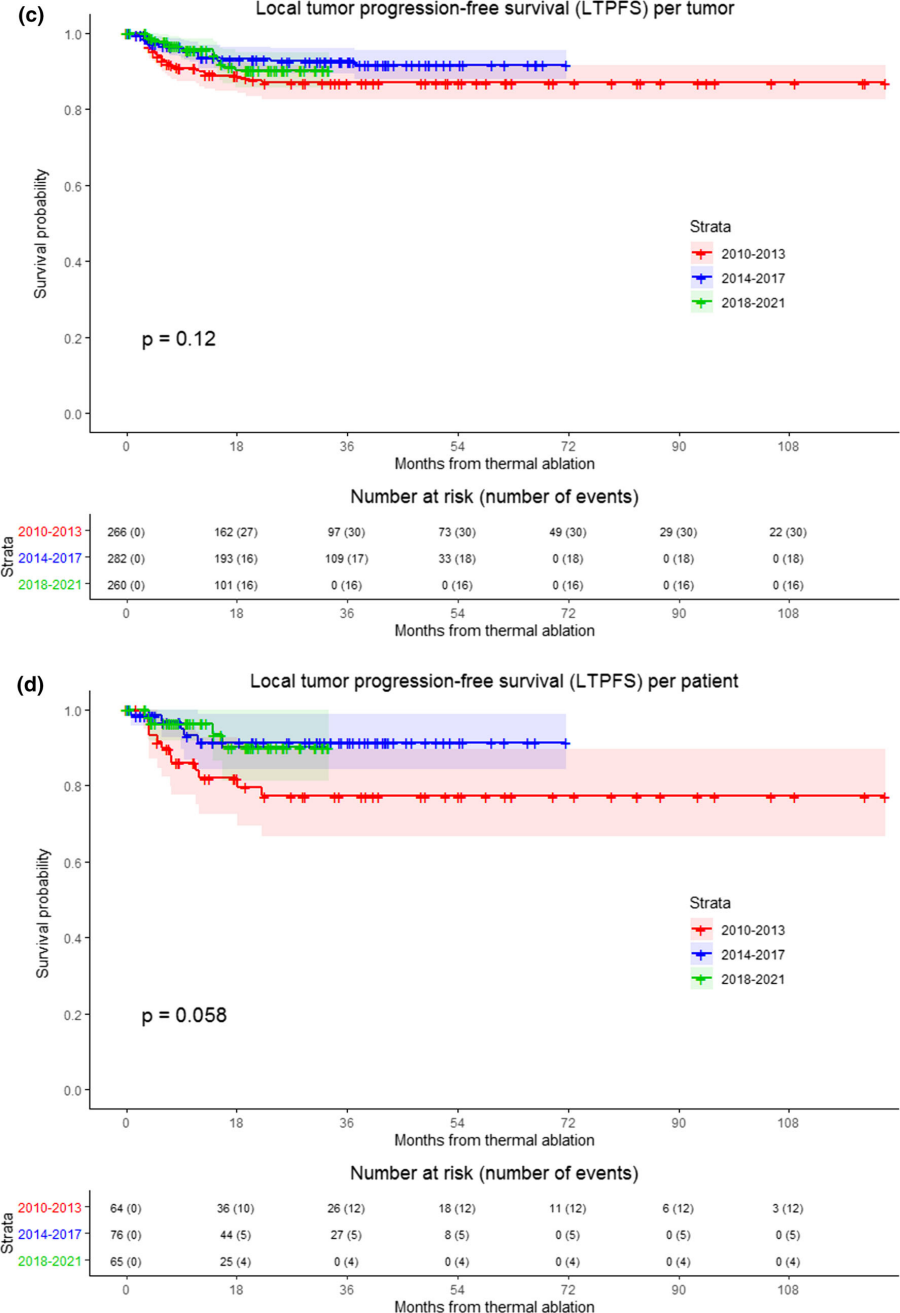

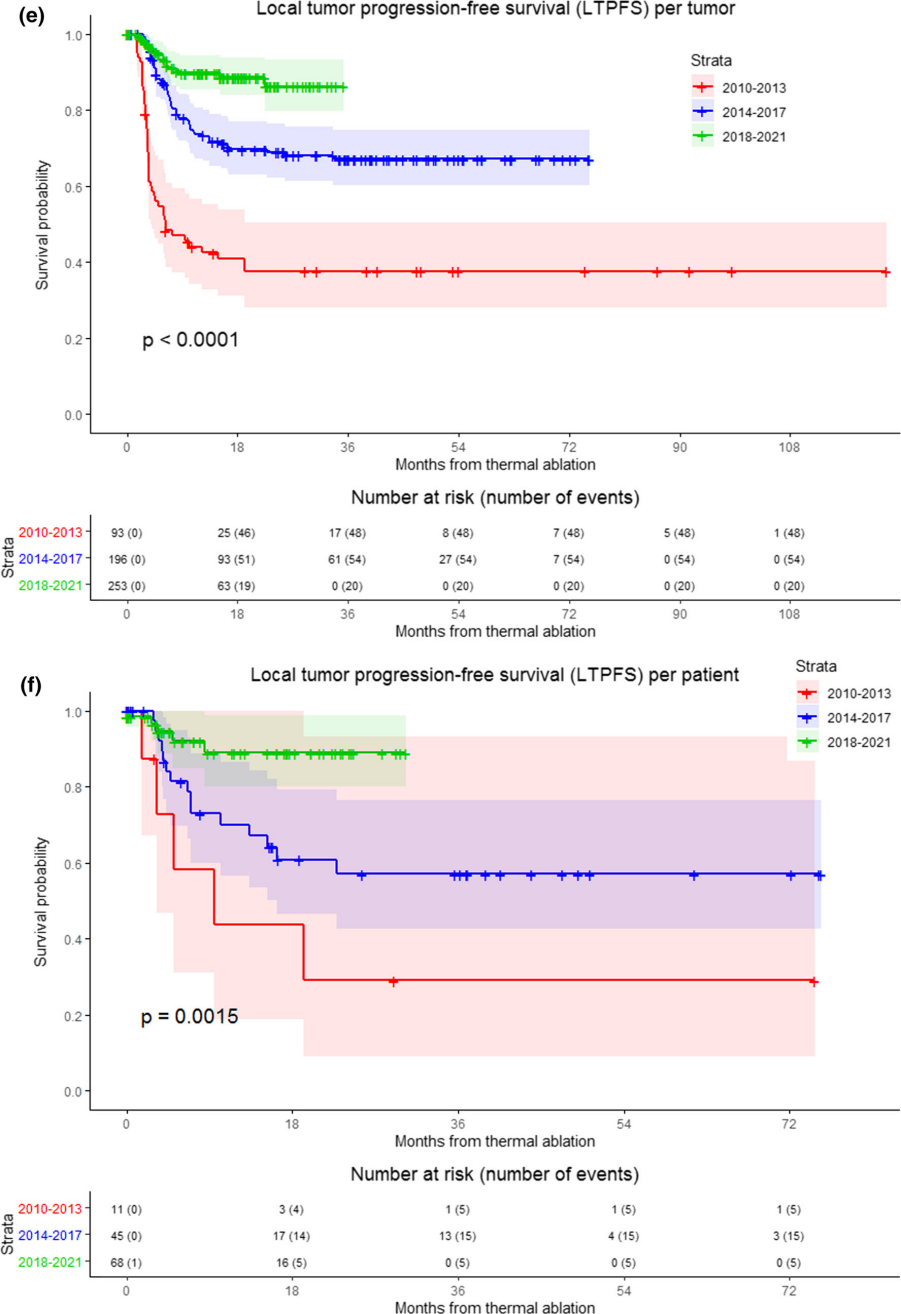
Kaplan–Meier survival curves indicating local tumor progression-free survival (LTPFS) per time frame and approach. **A**, **B** Analysis of open and percutaneous thermal ablation per treated tumor and per patient respectively, **C** and **D** patients treated with open ablation, analysis per treated tumor and per patient respectively, **E** and **F** patients treated with percutaneous ablation, analysis per treated tumor and per patient respectively. Numbers at risk correspond to either the amount of tumors or the number of patients. Overall comparison log-rank (Mantel–Cox) test is reported per graph. Death without local tumor progression (LTP) is censored (competing risk)

LTPFS per time frame is demonstrated in Fig. 3 (*P* < .0001). A per approach sub-analysis revealed LTP rates of 7.9% (64/808) for liver metastases treated with open ablation and 22.5% (122/542) for percutaneously ablated tumors, as shown in Fig. 4. The two-year LTPFS rate improved from 37.7% (2010–2013), to 69.0% (2014–2017) to 86.3% (2018–2021) (*P* < .0001) for patients treated with percutaneous ablation, while no temporal difference was found in LTPFS for patients treated with open ablation (87.1% vs 92.7% vs 90.2%, respectively; *P* = .12) (Fig. 4c-f; *P*-values in chart). Improvement in LTPFS was most remarkable after percutaneous ablation—as such, sub-analysis of all percutaneous procedures was performed to evaluate potential influencing factors. Procedure- and tumor-related characteristics of all percutaneous ablations are listed in Appendix 2a and 2b. Multivariable analysis revealed two factors associated with a superior LTPFS: Anesthetic management (HR 0.296 for propofol sedation and HR 0.978 for general anesthesia compared to midazolam + fentanyl sedation, *P* < .001). Adjusted HR of percutaneous ablations performed between 2014 and 2017 compared to 2010–2013 was 0.495 (95% CI (0.289–0.847) and 2018–2021 compared to 2010–2013 was 0.221 (95% CI 0.107–0.459) (*P* < .001).

### Complications and Length of Hospital Stay

Grade 1–5 complication rate in the entire cohort was 20.5% (111/541 procedures; Table 2). The severity of complications did not change over time (*P* = .404). The rate and severity of complications favored the percutaneous approach in all three cohorts (2010–2013, *P* = .069; 2014–2017, *P* = .129; 2018–2021, *P* = .020). Sub-analysis of procedures were thermal ablation was used solely (in other words without simultaneous resection or irreversible electroporation in case of open procedures), revealed no difference in complication rate between the three time frames (*P* = .406).

Overall procedure-related mortality was 1.5% (5/329) in the entire cohort. One patient deceased 7 days after combined liver resection and ablation due to massive pulmonary embolism (30-day mortality 0.4%; *n* = 1/329). Five others died from postoperative complications between 30 and 90 days: one due to massive portal thrombosis and multi-organ failure 5 weeks after combined percutaneous ablation and irreversible electroporation, and three due to abdominal abscesses and cardiopulmonary failure 8–9 weeks after combined liver resection and open ablation.

For open ablations, the mean length of hospital stay did not significantly differ between the three time frames (mean 6.9 days [SD 5.9]; *P* = .065). Mean hospitalization after percutaneous procedures was 1.4 days (SD 2.6) with no differences between the three cohorts (*P* = .054).

### Overall Survival

**Fig. 5:**
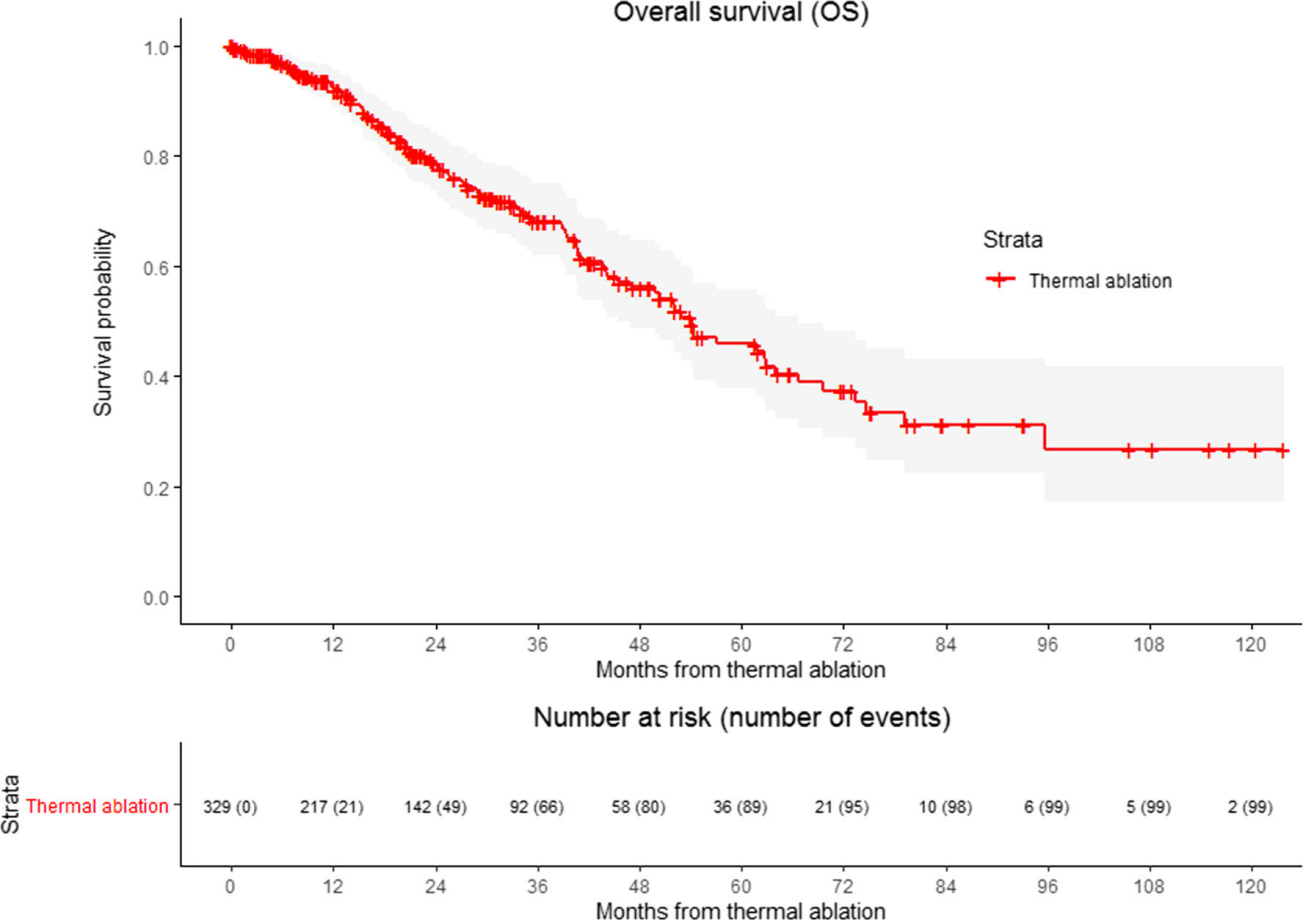
Kaplan–Meier survival curve of overall survival (OS) for patients treated with thermal ablation. Numbers at risk correspond to the number of patients

A total of 99 patients (30.1%) deceased during follow-up (Table 2). Of them, 93 died from disease progression. Survival probability after the first ablative treatment was 92.0%, 78.8%, 45.9% and 26.8% at 1, 3, 5 and 10 years, respectively (Fig. 5). For the entire cohort, the median OS after the first ablation procedure was 54.2 months; 52.0 months in the 2010–2013 cohort and 66.6 months in the 2014–217 cohort. The median OS for the latter cohort was not met. The median OS did not significantly improve over the last decade (*P* = .088), nor differed for patients treated by open or percutaneous ablation (*P* = .888).

## Discussion

Over the past decades, thermal ablation has become the standard treatment option to eradicate small unresectable CRLM (≤ 3 cm) and a fair alternative for deep-seated resectable CRLM that would otherwise require major hepatectomy [1, 2, 4]. Though advances in energy delivery in methods for precise probe placement and in ablation confirmation techniques have, often prematurely, been introduced as alleged improvements, our results underwrite technological progresses made over time. The improvement over time, in terms of LTPFS, especially for patients being treated with CT-guided percutaneous ablations, was the most remarkable finding in our study. OS did not significantly improve over the last 10 years. Whether this reflects an absent correlation between survival and local treatment failure, especially given the relative ease to repeat ablations, or the gradual acceptance to offer curative intent ablations to more complex cases with higher disease burden, remains unknown.

Results of this study compare well with OS and LTPFS data published in other recent series regarding thermal ablation of CRLM [1, 14, 35–39, 46, 47]. We have reached the point where the local tumor progression rate after percutaneous ablation has approached results following open ablation as well as following partial hepatectomy, as the most recent surgical series report R1/R2 rates varying from 12 to 46% [48–52]. Outcomes of this current cohort study are again underlining the necessity to conduct a randomized controlled trial comparing standard partial hepatectomy to its less invasive competitor thermal ablation for smaller-size resectable CRLM (≤ 3 cm). Although the phase III randomized LAVA trial (*ISRCTN52040363*) attempted to randomize high surgical risk CRLM patients to surgery or thermal ablation, recruitment feasibility was not established during the pilot stage, and therefore, the trial closed early without having gathered data regarding the primary endpoint two-year disease-free survival [53]. The interim results of the COLLISION trial (*NCT03088150*), presented at CIRSE 2021 and ECIO 2022, confirm thermal ablations’ superior safety profile, shorter hospital stay, equal to superior local control and similar OS compared to partial hepatectomy; the final results are eagerly awaited [54, 55]. Though a recent comparative analysis favored thermal ablation with regard to OS, LTPFS and eventual local control for small-size (≤ 3 cm), stereotactic body radiation therapy (SBRT) does challenge thermal ablation for intermediate-size (3-5 cm) CRLMs; the ongoing COLLISION-XL trial (*NCT04081168*) will hopefully provide clarity. [56].

Although speculative, the improvement over time, in terms of LTPFS, for patients being treated with percutaneous ablation should probably be contributed to (A) gained experience and (B) technological advancements made during the last decades. A multitude of minor improvements with regard to energy delivery spectrum, antenna and generator design (e.g., Thermosphere™ technology, multiple antennae systems or stereotactic navigation), anesthesia and breath-hold techniques, real-time image guidance (e.g., administration of intra-arterial contrast via an hepatic artery catheter) and the use of rigid and non-rigid image fusion and registration platforms allowing intraprocedural completion ablations seem to have led to this major quality improvement [6, 7, 22, 24–26, 31, 57–61].

Some limitations need to be addressed. The median follow-up period in the 2018–2021 cohort was sufficient (11.5 months), but inevitably lower compared to the earlier cohorts. This may have led to the situation where some patients in the latest cohort are still susceptible to developing LTP (immortality time bias), though this only applies to a small amount of tumors; as historically seen, the vast majority of LTPs are detected within the first 3–9 months following local treatment and a clear LTPFS plateau is reached after roughly 18 months follow-up (Fig. 2a) [9]. Reported study data were analyzed from prospectively kept records, and potential confounders were excluded by uni- and multivariable analyses, which does not fully guarantee that residual confounding has been eliminated. The fact that periprocedural chemotherapy regimens and follow-up imaging protocols did not change over time decreases the likelihood for residual bias. The lack of a comparison between laparoscopic and open ablated tumors could be a potential limitation as in certain cases the laparoscopic approach might be superior to the open approach in terms of safety and length of hospital stay. Due to technological advancements in energy delivery and reduced procedure time, MWA was gradually favored over RFA, even though previously published data showed no significant difference in terms of local disease control [6, 60, 61]. Nonetheless, the ablation modality need to be addressed as potential confounder. In addition, the specific ablation devices used in this study may render the comparative results as they do not necessarily represent all current day ablation systems. Although mutant RAS and BRAF status are known to be associated with LTP [47, 62], these tumor characteristics were not routinely measured over the last decade, resulting in high rates of missing data. Furthermore, it should be noted that the national guideline recommendations not routinely offer neo-adjuvant or adjuvant chemotherapy for locally treatable disease, what differs from several other countries and regions, and hence, it may be challenging to compare our results with series where patients were routinely offered (neo-)adjuvant systemic therapy [41]. However, the national guideline recommendations did not change over time and were actually re-established following the recent publication of two clinical trials of which one showed no difference in OS for perioperative chemotherapy (EORTC 40983) [63] and one showed an inferior OS for adding adjuvant chemotherapy (JCOG 0603) [64] .

In conclusion, the efficacy of percutaneous ablations for CRLM in terms of local tumor progression-free survival has improved remarkably over the last 10 years and seems to have approached oncological outcomes following open ablations. Over the last decade, no differences were found regarding length of hospital stay, rate and severity of complications, and overall survival. Given its minimal invasive character and shorter length of hospital stay, whenever feasible, percutaneous procedures may be favored over an open approach.

## Supplementary Information

Below is the link to the electronic supplementary material.

Supplementary file1 (DOCX 15 kb)

Supplementary file2 (DOCX 22 kb)

Supplementary file3 (DOCX 14 kb)
