# Supplementary material for: Improved Outcomes of Thermal Ablation for Colorectal Liver Metastases: A 10-Year Analysis from the Prospective Amsterdam CORE Registry (AmCORE)
Source: Cardiovasc Intervent Radiol. 2022 May 18;45(8):1074–89. doi: 10.1007/s00270-022-03152-9 (PMC9307533; doi:10.1007/s00270-022-03152-9)
Supplement: Supplementary file 1 — Supplementary file1 (DOCX 15 kb) [file 270_2022_3152_MOESM1_ESM.docx]

**Appendix 1: Ablation Method**

All patients were subjected to a routine check-up by the anesthesiologist. Before each procedure, patients were fasted for at least 6 hours.

**Anesthesia**

All open procedures were performed under general anesthesia, consisting of propofol (Diprivan®, AstraZeneca BV, Zoetermeer, the Netherlands), rocuronium (Esmeron®, Sandoz BV, Almere, the Netherlands), and remifentanyl (Ultiva®, Mylan BV, Amstelveen, the Netherlands] or sufentanil (Sufenta®, Janssen Pharmaceutica, Beerse, Belgium). Percutaneous procedures were performed under either general anesthesia, deep sedation (350-1000mg propofol [Diprivan®] plus either 200-700μg alfentanil [Rapifen®, Janssen Pharmaceutica, Beerse, Belgium] or 150-350μg remifentanyl [Ultiva®, Mylan BV, Amstelveen, the Netherlands], or moderate sedation (2.0-6.0 mg midazolam [Dormicum®, Roche BV, Woerden, the Netherlands] plus 100-300μg fentanyl [Durogesic®, Janssen Pharmaceutica, Beerse, Belgium]). Patients’ vitals were continuously monitored during each procedure. Regardless of the procedure, patients were admitted to the post-anesthesia care unit for observation. All patients stayed in the surgical ward for at least one night.

**Devices**

The equipment used in the study included the following systems: RF3000™ with LeVeen™ electrodes (Boston Scientific, Marlborough, Massachusetts, USA), Cool-tip™ RF ablation system (Medtronic-Covidien, Minneapolis, Minnesota, USA), Starburst® RF ablation system (AngioDynamics, Amsterdam, the Netherlands), Evident™ MW ablation system (Medtronic-Covidien, Minneapolis, Minnesota, USA), Emprint™ MW ablation system with Thermosphere™ Technology (Medtronic-Covidien, Minneapolis, Minnesota, USA) and Solero™ MW ablation system (AngioDynamics, Amsterdam, the Netherlands), which were used according to the manufacturer recommendations.

RFA and MWA were not simultaneously used during a procedure. Within our institution, MWA is generally the preferred modality for perivascular located tumors. For both CT fluoroscopy and CTHA guidance, preprocedural baseline images were obtained just prior to the actual procedure. The RFA device was set to automatically increase power to control impedance and avoid early roll-off. The MWA device was manually set to deliver 100W for 10 minutes.

**Procedure details**

All open ablations were guided by intra-operative ultrasound (IOUS) and performed by an interventional radiologist. All percutaneous procedures were performed in the ambulatory interventional oncology suite, which houses an angiography system (Azurion, Philips, Amsterdam, the Netherlands), CT scanner (SOMATOM Sensation or Drive, Siemens AG, München, Germany) and anesthetic facilities. Patients were positioned in a supine position with their head and spine aligned. Both arms are fixated above the head and tucked to the table to prevent brachial neuropathy. In case of dorsally located tumors (segment 6 or 7), patients were positioned in an oblique and lateral decubitus position. The table was secured in a stable position in order for the needle to remain in the appropriate plane.

CT fluoroscopy guidance

Conventional CT fluoroscopy was typically chosen in patients with contraindications (arterial stenosis or arteriosclerosis) for catheter placement and for well-visible tumors on (un)enhanced diagnostic CT. Baseline scan, just prior to the needle advancement, was obtained by administering 80cc of contrast agent (1:1 mixed bolus of contrast and saline; Xenetix 300, Guerbet SA, Villepinte, France) via a peripheral intravenous injection. Continuous radiation, with repetitive injection of 80cc contrast agent, was applied to advance and guide the needle into the tumor.

CT hepatic arteriography (CTHA) guidance

Patients were admitted to the angiography suite where the transarterial catheter was being inserted. The sheath was introduced in the right common femoral artery and the catheter was placed with the tip preferably in the common hepatic artery. A 4-F Cobra (Cordis Corporation, Bridgewater, New Jersey, USA) or 5-F Cobra (Cook, Bloomington, Indiana, USA) was used. In case of unstable positioning the tip was advanced in the proper hepatic artery, or in the left or right hepatic artery (depending on the location of the tumor[s]). Prior to the procedure, a baseline CTHA scan was performed for treatment planning and postprocedural image fusion.

A contrast bolus of 40cc (1:1 mixed bolus of contrast with saline; Xenetix 300) was injected into the arterial catheter at a flow rate of 5 cm^3^/s. Arterial phase, and mixed late arterial, early-to-portal venous phase CT images were acquired at 6 and 22 seconds, respectively. New tumors were being recognized by an enhancing ring with a hypoattenuating core or, in case of local tumor progression, by a typical incomplete enhancing ring. Along with introducing the electrode or antenna, repetitive small amounts of contrast (20cc, 1:1 mixed bolus) were injected to obtain real-time series of CTHA images in order to keep track of the position of the tip in relation to the target tumor and surrounding critical structures. When considered necessary, additional boluses of contrast (20cc) can be administered to allow for overlapping (completion) ablations.

The tip of the catheter may dislocate while the patient is being transferred from bed-to-bed. In that case, the tip may be positioned in the abdominal aorta to obtain an arteriogram without compromising the images. As a result, slightly more contrast needs to be injected (80cc, 1:1 mixed bolus with saline).

The arterial sheath was removed directly after the procedure by placing a hemostatic closure device at the insertion in the common femoral artery.

Ablation confirmation

Track ablation was routinely performed after each ablation to diminish bleeding and tumor seeding along the needle track. For ablation performed during an open approach, technical success was routinely checked by IOUS through multiple scansion planes. Per standard operating procedure, immediate postprocedural CT-images were acquired to assess for technical success after each percutaneous procedure. Intravenous contrast (80cc contrast agent, 1:1 mixed bolus) was used in case of CT fluoroscopy guidance; intra-arterial hepatic contrast (40cc, 1:1 mixed bolus) was administered in case of CTHA-guidance. Rigid fusion confirmation software ‘Syngo Fusion’ (Siemens Healthineers, Erlangen, Germany) was used to overlay pre- and postprocedural CT-images in order to assess immediate treatment success. In both circumstances incomplete ablated tumors were identified by persistent contrast enhancement at the tumor-site on postprocedural images. Complete ablation was defined as having reached circumferential coverage of the tumor plus a certain tumor-free margin by the ablation zone. The postprocedural CT-scan also served as recognizer of complications. In case of technical failure, an additional, overlapping ablation was performed to treat residual unablated viable tumor tissue.
