# Supplementary material for: Improved Outcomes of Thermal Ablation for Colorectal Liver Metastases: A 10-Year Analysis from the Prospective Amsterdam CORE Registry (AmCORE)
Source: Cardiovasc Intervent Radiol. 2022 May 18;45(8):1074–89. doi: 10.1007/s00270-022-03152-9 (PMC9307533; doi:10.1007/s00270-022-03152-9)
Supplement: Supplementary file 2 — Supplementary file2 (DOCX 22 kb) [file 270_2022_3152_MOESM2_ESM.docx]

**Appendix 2a. Clinical characteristics of percutaneous procedures only.**

|  |  | **Total** | **2010-2013** | **2014-2017** | **2018-2021** | ***P* value** |
| --- | --- | --- | --- | --- | --- | --- |
| **Patient-related characteristics** |  | **N=124** | **N=11** | **N=45** | **N=68** |  |
| Gender | Male  Female | 82  42 | 8  3 | 34  11 | 40  28 | .164 ^a^ |
| Age, years * |  | 66.5 (10.6) | 65.9 (11.5) | 65.3 (10.5) | 67.4 (10.5) | .587 ^b^ |
| ASA physical status | 1  2  3  Unknown | 9  82  30  3 | 2  4  3  2 | 5  32  8  - | 2  46  19  1 | .109 ^a^ |
| Comorbidities | None  Minimal  Major  Unknown | 58  43  21  2 | 4  3  2  2 | 25  14  6  - | 29  26  13  - | .729 ^a^ |
| BMI (kg/cm^2^) * |  | 26.1 (4.0) | 24.4 (4.4) | 26.0 (4.8) | 26.4 (5.1) | .489 ^b^ |
| **Disease-related characteristics** |  |  |  |  |  |  |
| **Clinical Risk Score (CRS)** | 0-2  ≥3  Unknown | 61  15  48 | 3  1  7 | 15  5  25 | 42  9  16 | .771 ^a^ |
| Diagnosis of CRLM | Synchronous  Metachronous  Unknown | 46  64  14 | 3  5  3 | 11  24  10 | 32  35  1 | .274 ^a^ |
| Primary tumor location | Right-sided  Left-sided  Rectum  Unknown | 24  66  33  1 | 1  4  5  1 | 9  27  9  - | 14  35  19  - | .397 ^a^ |
| RAS status | RAS wildtype  RAS mutation  Unknown | 10  7  107 | 0  0  11 | 4  0  41 | 6  7  55 | .056 ^a^ |
| BRAF V600 status | BRAF wildtype  BRAF mutation  Unknown | 17  0  107 | 0  0  11 | 4  0  41 | 13  0  55 | NA |
| MSS/MSI status | MSS  MSI  Unknown | 23  1  100 | 0  0  11 | 6  0  39 | 17  1  50 | .555 ^a^ |
| **Procedure-related characteristics** |  | **N=309** | **N=53** | **N=121** | **N=135** |  |
| Situation | Thermal ablation alone  Simultaneous partial hepatectomy  Simultaneous IRE | 286  NA  23 | 53  NA  - | 109  NA  12 | 124  NA  11 | .150 ^a^ |
| Induction chemotherapy | No  Yes | 229  80 | 40  13 | 90  31 | 99  36 | .952 ^a^ |
| No. of locally treated tumors | 1-3  ≥4 | 278  31 | 47  6 | 113  8 | 118  17 | .266 ^a^ |
| Anesthesia technique | General anesthesia  Midazolam + Fentanyl sedation  Propofol sedation  Unknown | 85  68  152  4 | 32  19  -  2 | 21  49  50  1 | 32  -  102  1 | <.001 ^a^ |
| Image-guidance technique | Conventional (CT fluoroscopy)  CT hepatic arteriography | 70  239 | 37  16 | 20  101 | 13  122 | <.001 ^a^ |
| Ablation modality | Radiofrequency  RF3000™, LeVeen™  Cool-tip™  Starburst® (RITA®)  Unknown  Microwave  Evident™  Solero™  Emprint™ with Thermosphere™  Unknown  Unknown | 97  86  5  4  2  210  19  6  180  5  2 | 40  35  2  3  -  7  5  0  4  2  2 | 55  51  3  1  -  66  12  4  49  1  - | 2  -  -  -  2  133  2  2  127  2  - | <.001 ^a^ |
| **Tumor-related characteristics** |  | **N=542** | **N=93** | **N=196** | **N=253** |  |
| Diameter, mm * |  | 18.0 (11.7) | 21.3 (13.6) | 17.0 (12.0) | 17.7 (15.0) | .014 ^b^ |
| Size, mm | Small (1-30)  Intermediate (31-50)  Large (>50)  Unknown | 436  64  11  31 | 63  12  5  13 | 157  23  4  12 | 216  29  2  6 | .053 ^a^ |
