# Supplementary material for: Improved Outcomes of Thermal Ablation for Colorectal Liver Metastases: A 10-Year Analysis from the Prospective Amsterdam CORE Registry (AmCORE)
Source: Cardiovasc Intervent Radiol. 2022 May 18;45(8):1074–89. doi: 10.1007/s00270-022-03152-9 (PMC9307533; doi:10.1007/s00270-022-03152-9)
Supplement: Supplementary file 3 — Supplementary file3 (DOCX 14 kb) [file 270_2022_3152_MOESM3_ESM.docx]

**Table 3. Factors associated with local tumor progression-free survival identified by univariable and multivariable Cox regression analyses from the time of the first intervention to local tumor progression, of percutaneous procedures only.**

|  | | **Univariable Analysis** | | **Multivariable Analysis** | |
| --- | --- | --- | --- | --- | --- |
|  |  | **HR (CI)** | ***P*-value** | **HR (CI)** | ***P*-value** |
| Timeframe | 2010-2013 | Reference | **<.001** | Reference | **<.001** |
|  | 2014-2017 | 0.330 (.0224-0.488) |  | 0.495 (0.289-0.847) |  |
|  | 2018-2021 | 0.123 (0.073-0.208) |  | 0.221 (0.107-0.459) |  |
| **Procedure-related factors** | | | | | |
| Anesthesia | Dormicum | Reference | **<.001** | Reference | **<.001** |
|  | Propofol  General anesthesia | 0.181 (0.108-0.303)  0.977 (0.670-1.475) |  | 0.296 (0.160-0.546)  0.978 (0.615-1.555) |  |
| Catheter-guided | No | Reference | **<.001** | Reference | **.293** |
|  | Yes | 0.418 (0.287-0.607) |  | 0.768 (0.469-1.256) |  |
| Modality | RFA | Reference | **<.001** | Reference | **.201** |
|  | MWA | 0.388 (0.272-0.555) |  | 1.352 (0.852-2.145) |  |
| **Tumor-related factors** | | | | | |
| Size of metastasis (mm) | Small (1-30) | Reference | **.015** | Reference | **.101** |
|  | Intermediate (31-50) | 1.617 (0.982-2.665) |  | 1.427 (0.860-2.368) |  |
|  | Large (>50) | 3.759 (1.517-9.315) |  | 2.533 (0.895-7.166) |  |
